# Supplementary material for: STIM1/2 maintain signaling competence at ER-PM contact sites during neutrophil spreading
Source: J Cell Biol. 2025 Mar 21;224(5):e202406053. doi: 10.1083/jcb.202406053 (PMC11927589; doi:10.1083/jcb.202406053)
Supplement: Table S1 — shows the effects of soluble molecules and substrates on Ca2+ signals during neutrophils spreading. [file jcb_202406053_tables1.docx]

|  |  |  | **% active cells (mean ± SEM)** | | | **Integrated Response (mean ± SEM)** | | |
| --- | --- | --- | --- | --- | --- | --- | --- | --- |
| **Agonist** | N= | CA Response | Pre | Post | p | Pre | Post | p |
| **DMSO** | 25 | N/A | 70.39 ± 4.71 | 65.09 ± 5.90 | 0.21 | 5.76 ± 1.20 | 4.87 ± 0.63 | 0.48 |
| **fMIVIL** | 38 | 35.81 ± 7.22 | N/A | N/A | - | N/A | N/A | - |
| **Tg** | 22 | 49.54 ± 8.05 | N/A | N/A | - | N/A | N/A | - |
| **LPS** | 15 | 35.58 ± 4.59 | 60.13 ± 6.10 | 62.50 ± 7.08 | 0.65 | 5.39 ± 1.04 | 5.04 ± 1.25 | 0.71 |
| **LTB4** | 7 | 26.26 ± 7.06 | 69.50 ± 6.50 | 90.00 ± 2.00 | N/A | 7.44 ± 2.22 | 5.90 ± 1.23 | N/A |
| **PMA** | 3 | N/A | 69.00 ± 13.43 | 43.33 ± 18.78 | 0.51 | 4.42 ± 0.92 | 2.49 ± 1.21 | 0.43 |
| **C5a** | 9 | N/A | 70.00 ± 5.42 | 62.33 ± 7.11 | 0.30 | 4.83 ± 0.67 | 4.46 ± 1.34 | 0.81 |
| **TNF** | 5 | N/A | 72.25 ± 11.08 | 85.80 ± 3.92 | 0.45 | 7.10 ± 2.29 | 7.5 ± 1.38 | 0.60 |
| **MSU** | 7 | N/A | 54.33 ± 9.59 | 75.67 ± 13.47 | 0.13 | 3.89 ± 2.15 | 12.18 ± 2.98 | 0.03 |
| **Paf** | 3 | 19.25 ± 1.93 | N/A | N/A | - | N/A | N/A | - |

*Some compounds displayed mixed effects with either “Response” or “flickers”. Where n<3 p = N/A

|  |  |  | **% active cells (mean ± SEM)** | | | **Integrated Response (mean ± SEM)** | | |
| --- | --- | --- | --- | --- | --- | --- | --- | --- |
| **Blockers** | N= | CA Response | Pre | Post | p | Pre | Post | p |
| **EGTA** | 6 | N/A | 55.33 ± 13.56 | 42.50 ± 13.92 | 0.40 | 7.46 ± 3.89 | 3.37 ± 1.48 |  |
| **U73** | 4 | N/A | 74.00 ± 12.64 | 36.75 ± 9.64 | 0.004 | 4.15 ± 0.60 | 1.75 ± 0.52 | 0.047 |
| **U75+LY2** | 4 | N/A | 81.00 ± 7.12 | 59.25 ± 10.00 | 0.12 | 6.34 ± 1.45 | 4.10 ± 1.09 | 0.10 |
| **TRAM34** | 3 | N/A | 81.00 ± 7.10 | 77.00 ± 4.73 | 0.68 | 28.72 ± 9.98 | 19.98 ± 7.44 | 0.46 |
| **GSK79** | 5 | N/A | 74.20 ± 10.97 | 75.20 ± 4.82 | 0.93 | 6.44 ± 2.15 | 7.77 ± 2.80 | 0.67 |
| **GSK48** | 2 | N/A | 75.00 ± 25.00 | 51.00 ± 16.00 | N/A | 17.61 ± 10.82 | 6.87 ± 1.21 | N/A |
| **DPI** | 4 | N/A | 41.75 ± 14.48 | 26.75 ± 7.33 | 0.20 | 1,67 ± 0.58 | 3.51 ± 1.43 | 0.18 |
| **TAK24** | 7 | +/- | 71.86 ± 7.22 | 57.71 ± 11.65 | 0.40 | 6.50 ± 1.02 | 4.39 ± 1.06 | 0.15 |
| **Gd** | 4 | N/A | 69.75 ± 6.40 | 61.25 ± 8.47 | 0.19 | 5.09 ± 1.77 | 2.53 ± 0.53 | 0.26 |
| **AbA9** | 2 | N/A | 26.50 ± 13.50 | 45.00 ± 5.00 | N/A | 9.33 ± 2.54 | 8.33 ± 0.57 | N/A |
| **MRS 2578** | 2 | + | 44.50 ± 11.50 | 88.00 ± 12.00 | N/A | 8.91 ± 2.77 | 43.41 ± 24.92 | N/A |

| **Substrates** | **N=** | **% active cells (mean ± SEM)** | **Integrated Response (mean ± SEM)** |
| --- | --- | --- | --- |
| **Glass (naked)** | 1 | 92.00 ± 0.00 | 10.82 ± 0.00 |
| **PBS** | 2 | 62.00 ± 9.00 | 7.01 ± 1.63 |
| **PLL** | 2 | 77.00 ± 6.00 | 5.94 ± 1.09 |
| **BSA** | 2 | 60.00 ± 7.00 | 5.54 ± 0.45 |
| **Vitronectin** | 1 | 81.00 ± 0.00 | 15.45 ± 0.00 |
| **Fibronectin** | 2 | 65.00 ± 22.50 | 6.24 ± 0.39 |
| **RPMI** | 2 | 20.50 ± 10.50 | 4.93 ± 0.81 |
| **FCS** | 2 | 66.50 ± 11.50 | 5.83 ± 1.67 |
