# Supplementary figures and images for: STIM1/2 maintain signaling competence at ER-PM contact sites during neutrophil spreading
Source: J Cell Biol. 2025 Mar 21;224(5):e202406053. doi: 10.1083/jcb.202406053 (PMC11927589; doi:10.1083/jcb.202406053)

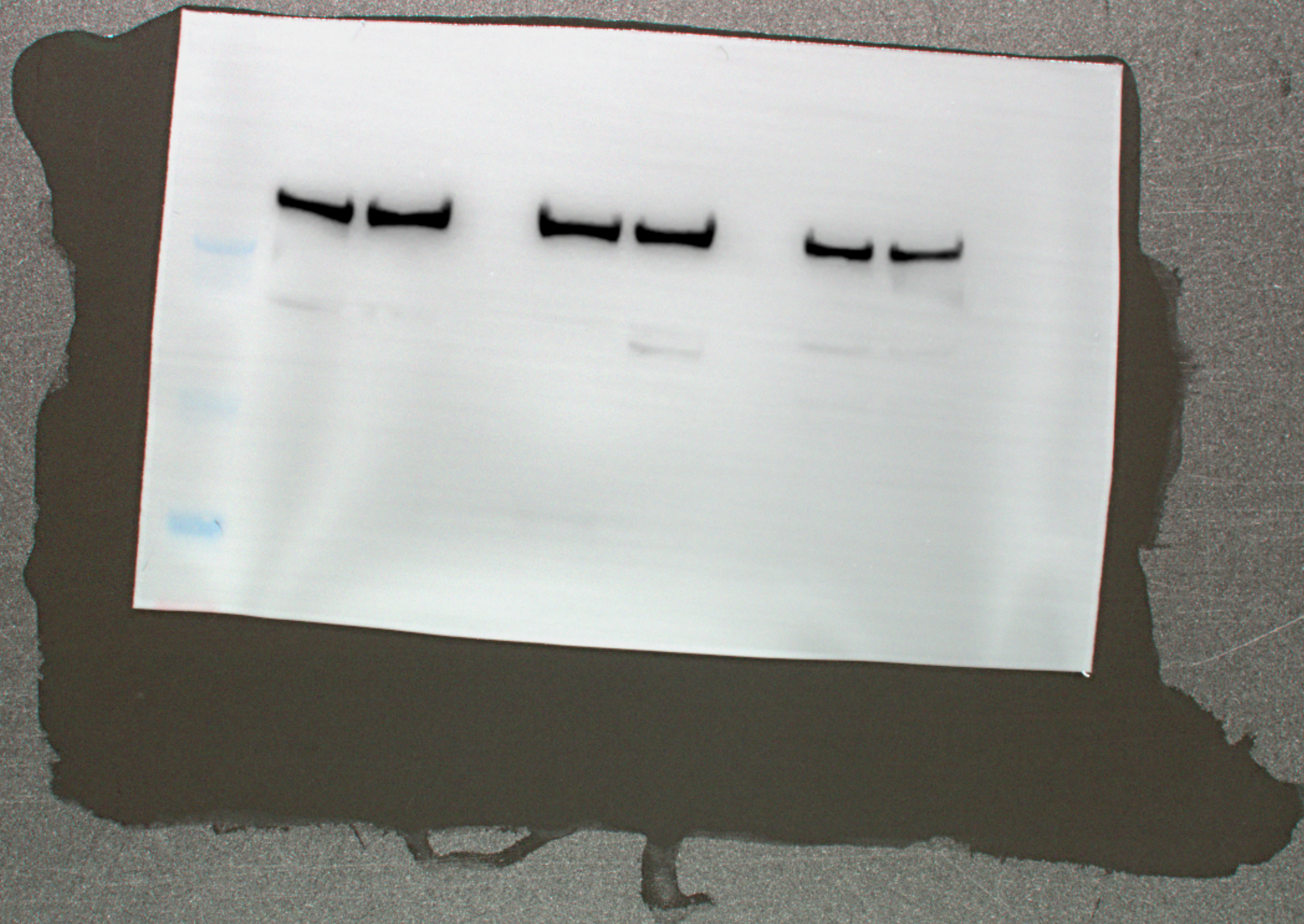

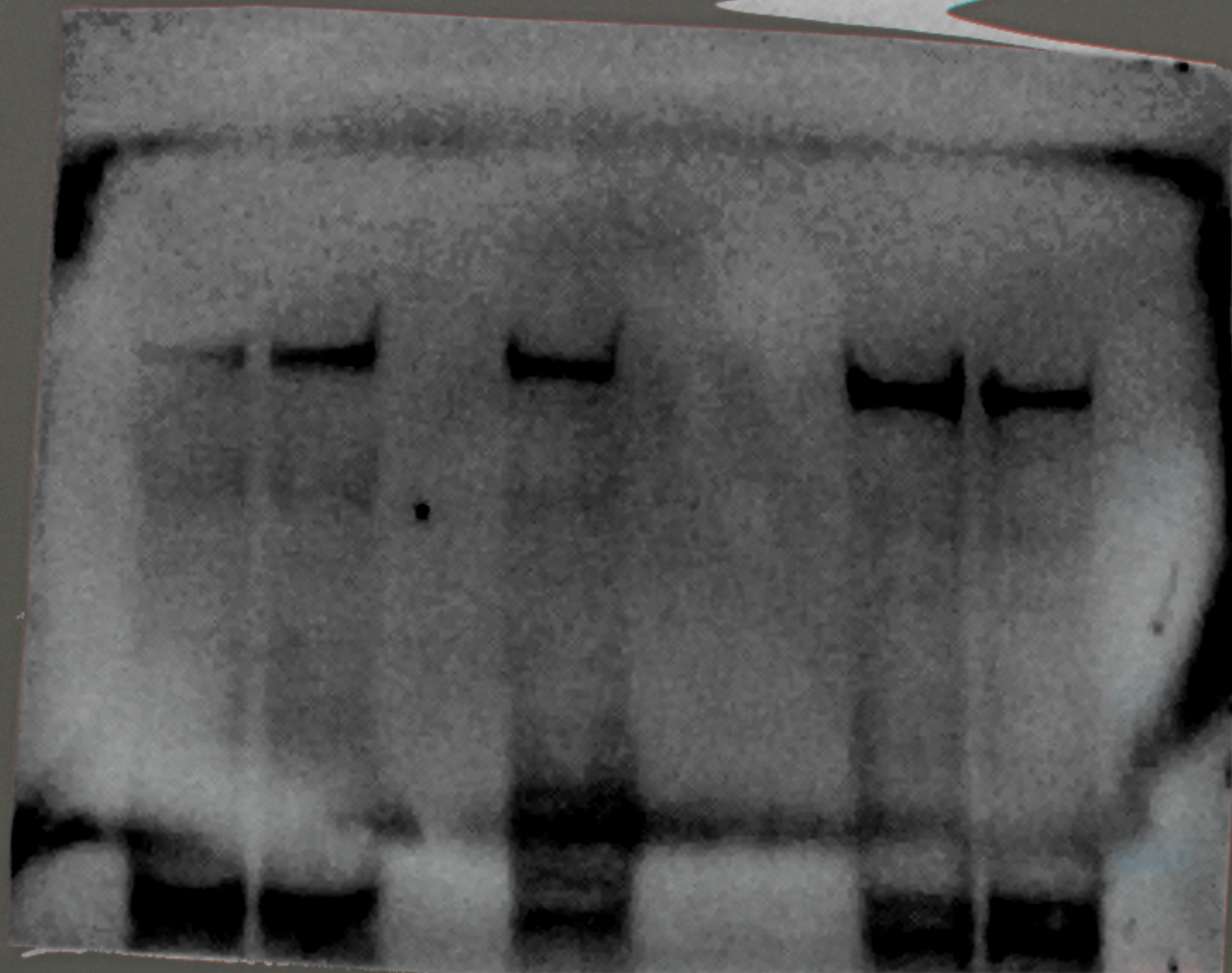

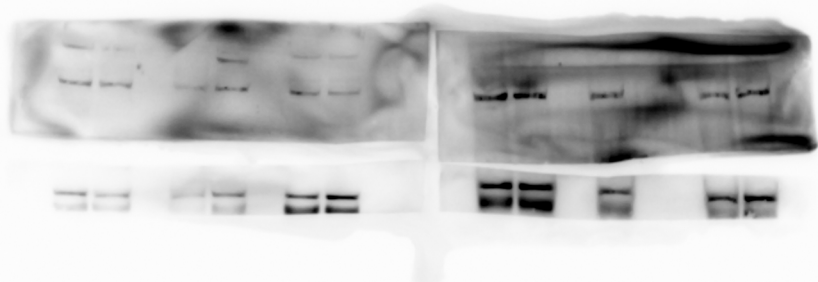

Supplement: SourceData F6 — is the source file for Fig. 6. [file jcb_202406053_sourcedataf6.pdf]
